# Biological Difference Between Epstein–Barr Virus Positive and Negative Post-transplant Lymphoproliferative Disorders and Their Clinical Impact

**DOI:** 10.3389/fonc.2020.00506

**Published:** 2020-05-08

**Authors:** Valeria Ferla, Francesca Gaia Rossi, Maria Cecilia Goldaniga, Luca Baldini

**Affiliations:** ^1^Hematology Division, IRCCS Ca' Granda-Maggiore Policlinico Hospital Foundation, Milan, Italy; ^2^University of Milan, Milan, Italy

**Keywords:** post-transplant lymphoproliferative disorders, Epstein–Barr virus, next-generation sequencing, microRNA, gene expression profile, tumor microenvironment

## Abstract

Epstein–Barr virus (EBV) infection is correlated with several lymphoproliferative disorders, including Hodgkin disease, Burkitt lymphoma, diffuse large B-cell lymphoma (DLBCL), and post-transplant lymphoproliferative disorder (PTLD). The oncogenic EBV is present in 80% of PTLD. EBV infection influences immune response and has a causative role in the oncogenic transformation of lymphocytes. The development of PTLD is the consequence of an imbalance between immunosurveillance and immunosuppression. Different approaches have been proposed to treat this disorder, including suppression of the EBV viral load, reduction of immune suppression, and malignant clone destruction. In some cases, upfront chemotherapy offers better and durable clinical responses. In this work, we elucidate the clinicopathological and molecular-genetic characteristics of PTLD to clarify the biological differences of EBV(+) and EBV(–) PTLD. Gene expression profiling, next-generation sequencing, and microRNA profiles have recently provided many data that explore PTLD pathogenic mechanisms and identify potential therapeutic targets. This article aims to explore new insights into clinical behavior and pathogenesis of EBV(–)/(+) PTLD with the hope to support future therapeutic studies.

## Introduction

The World Health Organization (WHO) classification of lymphoid malignancies considers four major diagnostic post-transplant lymphoproliferative disorder (PTLD) categories: early lesions, polymorphic PTLD that could be either polyclonal or monoclonal, Hodgkin lymphoma (HL), and monomorphic PTLD of which diffuse large B-cell lymphoma is most common (1).

PTLD can occur in 20% of hematopoietic stem cell (HSC) and solid organ transplant (SOT) recipients.

PTLD is associated with Epstein–Barr virus (EBV) infection in 60–80% of cases. In EBV infection in immunocompetent (IC) hosts, the virus forms an episome in latently infected B cells (2, 3). In post-transplant patients, immunosuppression causes T-cell inhibition with a consequent lack of T-cell modulation on B-cell proliferation. In particular, when an EBV(–) patient receives an EBV(+) transplant graft, immunosuppression causes uncontrolled proliferation of EBV-transformed B cells, which contributes to the development of PTLD (2, 4, 5).

The pathogenesis of EBV-PTLD is currently unclear; different hypotheses have been suggested as possible pathogenic mechanisms of these EBV-PTLD, such as chronic immune triggering by the graft, hit-and-run EBV infection (EBV induces chromosomal aberrations in cell genome and might be lost during malignant cell division), and other infectious agents [e.g., human herpesvirus 5, 6, or 8; (6–11)]. However, there is limited evidence supporting these hypotheses (Table 1).

**Table 1:** Major risk factors in the development of PTLD.

**Risk factors for PTLD**		
Infectious etiologies	EBV, especially when EBV(–) recipients received a transplant graft from EBV(+) donor. Mismatch for CMV, HCV, and HHV-8, especially when they coincided with EBV infection.	(5, 12)
Age and race	Ages <10 and >60 years. Race: White transplant patients > Blacks.	(13, 14)
Immunosuppressive therapy	The degree, duration, and type of immunosuppression (in particular, anti-thymocyte globulin, calcineurin inhibitors, anti-CD3, tacrolimus, and cyclosporine)	(15, 16)
HSCT/SOT-related factor	SOT types (multi-organ and intestinal transplants have an increasing risk than have lung transplants > heart transplants > liver transplants > pancreatic transplants > kidney transplants). HLA mismatch in HSCT (haploidentical transplants have an increasing risk than have unrelated donor > umbilical cord transplant > HLA-identical related). Type of GVHD prophylaxis, T-cell depletion has the highest risk. Severity of GVHD transplant.	(16–19)
Genetic factors	Polymorphisms in cytokine genes. Recipient HLA, donor polymorphisms.	(20, 21)

*EBV, Epstein–Barr virus; CMV, cytomegalovirus; HCV, hepatitis C; HHV, human herpesvirus; HSCT, hematopoietic stem cell transplant; HLA, human leukocyte antigen; GVHD, graft-vs.-host disease; SOT, solid organ transplant*.

Clinically, there are differences between EBV(–) and EBV(+) PTLD. In particular, it has been described that EBV(–) PTLD arises later, after years of transplantation, whereas EBV(+) cases arise earlier, generally after months. Furthermore, EBV RNA is detected in early and polymorphic lesions, typical lesions early after transplantation.

In the literature, contradictory data are described regarding the diversity of prognosis between the EBV(+) and EBV(–) cases; in particular, the international multicenter prospective phase 2 PTLD-1 trial found no association with overall survival and EBV status [(22, 23); Table 2].

**Table 2:** Clinical aspects of EBV(+)/(–) PTLD.

**Clinical aspects**	**EBV(+)/(–) PTLD**	**References**
Incidence	55–65% of PTLD is associated with EBV infection.	(21, 24)
Clinical presentation	EBV(–) occur later (years) than does EBV(+) PTLD (months). EBV(–) present more often as monomorphic PTLD.	(25)
Prognosis	Controversial results in literature about the different prognoses of EBV(+)/(–) PTLD.	(22)
Therapy and prospective	EBV(+) and EBV(–) PTLD have the same therapy. Specific immunotherapies for EBV(+) PTLD have been proposed, for example, adoptive T-cell transfer, immune checkpoint inhibitors, and antiviral therapy.	(23, 25)

*EBV, Epstein–Barr virus; PTLD, post-transplant lymphoproliferative disorder*.

From a therapeutic point of view, EBV(+) and EBV(–) PTLD have the same therapy; the only difference is regarding the EBV-specific adoptive immunotherapy.

Many studies have tried to investigate the genomic differences between the IC-DLBCL, EBV(+), and EBV(–) PTLD. What emerged was that EBV(–) PTLD has a genomic profile very similar to that of IC-DLBCL and a much greater biological complexity than EBV(+) PTLD (26–29).

Furthermore, it has been shown that EBV(+) PTLD, in addition to having a different genomic profile, has different genetic and tumor microenvironment alterations compared with those of EBV(–) PTLD (30–32).

Furthermore, EBV infection may alter the microRNA expression in B lymphocytes. MicroRNA is an important transcriptional and post-transcriptional regulator of gene expression.

In PTLD, EBV(+), B-cell lymphoma revealed different microRNA profiles, compared with normal B cells or EBV lymphoblastoid cell lines generated *in vitro* (33, 34).

These considerations seem to suggest that the pathogenesis of EBV(–) PTLD is to be considered much more similar to that of IC-DLBCL and that it is less influenced by post-transplantation factors. However, despite these differences, the fact that some EBV(–) PTLD respond well to reduction of immunosuppression similarly to EBV(+) PTLD remains to be clarified (35). Certainly, these studies seem to offer theoretical support for future therapeutic studies in EBV(+) and EBV(–) PTLD that appear to have a different pathogenesis.

## The Genomic Landscape of Epstein–Barr Virus Positive and Negative Post-Transplant Lymphoproliferative Disorders

In this work, we want to illustrate the genomic complexity of EBV(+) and EBV(–) PTLD through the integration of different genomic approaches that have significantly improved our understanding of the genetic landscape of these disorders (Table 3).

**Table 3:** Genomic characterization of EBV(+) and EBV(–) PTLDs through different technologies approaches.

**Genomic approach**	**EBV(+)/EBV(–) PTLD**	**References**
CGH FISH WGP SNP NGS	The most common copy number aberration in EBV(+) PTLD is the gain/amplification of 9p24, whereas in EBV(–) PTLD, it includes gain of 3/3q and 18q, loss of 6q23/TNFAIP3, and loss of 9p21/CDKN2A TP53 mutations were more frequent in EBV(–) PTLD than EBV(+) PTLD and IC-DLBC. Compared with EBV(+) PTLD, EBV(–) PTLD and IC-DLBC have more frequent gene mutations associated with the NF-κB pathway. EBV(+) PTLD has a constitutive activation of the PI3K/Akt/mTOR pathway.	(36) (26) (27) (31) (29) (37)
**TRANSCRIPTIONAL APPROACH**		
GEP MicroRNA expression	EBV(–) and EBV(+) PTLD demonstrated different GFP especially gene involved in inflammation and immune response pathway profile. EBV(+) PTLD has a suppressed expression of microRNA-194.	(38) (30) (31) (33)

*CGH, comparative genomic hybridization; FISH, fluorescence in situ hybridization; WGP, whole-genome prediction; SNP, single-nucleotide polymorphism; NGS, next-generation sequencing; IC-DLBC, immunocompetent diffuse large B cell; GEP, gene expression profiling; NF-κB, nuclear factor-κB*.

## Molecular Characterization Through a Genomic Approach

Poirel et al. (36) studied PTLD cases with comparative genomic hybridization (CGH) and fluorescence *in situ* hybridization (FISH). The overall incidence of chromosomal imbalances was described in half of PTLD cases, even in the polymorphic category. Latent EBV infection was found in the lesions of three quarters of cases. Non-random losses were 17p13; 1p36, 4q; and 17q23q25, Xp. The gains of 8q24, 3q27, 2p24p25, 5p, 9q22q34, 11, 12q22q24, 14q32, 17q, and 18q21 were the most frequent. Three amplifications −4p16, 9p22p24, and 18q21q23–were detected. FISH has confirmed the involvement of Bcl2 in this latter imbalance. Chromosomal imbalances tended to be more complex in EBV(–) cases than in EBV(+) cases. The identification of chromosomal regions non-randomly involved in lymphomagenesis supports the role of candidate genes to be identified by a combined approach using gene expression profiling (GEP) and CGH array.

In order to improve PTLD pathogenesis understanding, Rinaldi et al. studied recurrent lesions revealed by whole-genome profiling analysis (26). The most common gains in IC-DLBCL were chromosome 3q, 7q, 12, and 18q and in PTLD were chromosomes 5p and 11p. The most common losses in IC-DLBCL were chromosome 12p and in PTLD were 6q, 17p, 1p, and 9p. DNA loss did not always match with loss of heterozygosity (LOH), and uniparental disomy seems to target chromosome 10 in PTLD. They found small deletions and gains involving BCL2 and PAX5 and ZDHHC14 (known gene). These data show that PTLD, at a lower frequency, shares common genetic aspects with IC-DLBCL. 9p13 amplification supports the importance of PAX5 in PTLD pathogenesis. Different DNA copy number and LOH patterns support the hypothesis that uniparental disomy can have a role in lymphomagenesis.

High-density genome-wide single-nucleotide polymorphism (SNP)-based arrays were used by Rinaldi et al. (27) to compare PTLD with IC-DLBCL and to compare EBV(+) with EBV(–) PTLD. In PTLD, the more frequently deleted loci were small interstitial deletions targeting FRA1B, FRA2E, and FRA3B fragile sites. PTLD presents typical and different aberrations than does IC-DLBCL: the deletions at 2p16.1 (FRA2E), lack of del(13q14.3) (MIR15/MIR16), and copy neutral LOH affecting 6p MHC. EBV(+) PTLD presented less recurrent lesions than did EBV(–) PTLD, including a gain of 7p, del(4q25–q35), and gains of 7q, 11q24–q25.

Menter et al. (29) investigated PTLD through next-generation sequencing (NGS) using the Ion Torrent platform. Nuclear factor-κB pathway-related genes had fewer mutations in EBV(+) PTLD compared with IC-DLBCL. Moreover, in PTLD, compared with IC-DLBCL, TP53 was more frequently mutated, whereas ATM and B2M mutations were absent. TP53 mutations were more frequent in EBV(–) PTLD. Mutations in DNA damage control and immune-surveillance genes are different in PTLD with respect to IC-DLBCL. EBV seems to have a role in the different mutational pattern.

## Molecular Characterization Through a Transcriptional Approach

Through gene expression analysis, Morscio et al. (38) and Craig et al. (30) showed that EBV(+) and EBV(–) PTLD have different microenvironment and gene expression profiles. They also demonstrated that EBV(–) PTLD and IC-DLBCL are biologically similar.

Through array comparative genome hybridization (aCGH) analysis, Ferreiro et al. (31) studied at genomic and transcriptomic levels EBV(+) PTLD, EBV(–) PTLD, and IC-DLBCL.

EBV(+) PTLD had a different CNA pattern as compared with EBV(–) PTLD and a lower genomic imbalance.

Moreover, EBV(+) PTLD showed distinct aCGH profiles with only one recurrent imbalance with EBV(–) PTLD. On the other hand, EBV(–) PTLD displayed similar recurrent aberrations (gain of 3/3q and 18q and loss of 6q23/TNFAIP3 and 9p21/CDKN2A) as compared with IC-DLBCL. These findings support the concept of a biological relationship between both conditions.

9p24.1 gain/amplification was the most frequent aberration in EBV(+) PTLD targeting PDCD1LG2/PDL2. These genes encode immunomodulatory programmed cell death ligands (39).

In lymphoproliferative disorder, particularly in primary mediastinal B-cell lymphoma, classical HL, and primary central nervous system lymphoma, 9p24.1 is a common copy number gain. The consequence of this alteration is an increase of PDL1 and PDL2 and their induction by *JAK2* (40–43).

An upregulation of PDL1 was described in the majority of EBV(+) lymphomas, including PTLD (44–46). PDL1/2 signal regulates immune defenses against pathogens and T-cell tolerance/T-cell activation through the PD-1 receptor (47).

**Figure 1:**
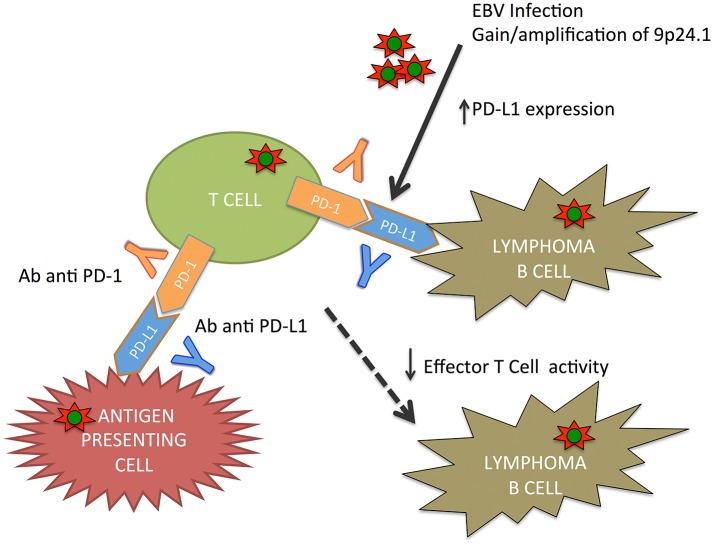
PD-1/PD-L1 pathways in EBV(+) PTDL.

Green et al. (44) demonstrated an alternative activation mechanism of PDL1 in classical HL and EBV(+) lymphoma, in which EBV latent membrane protein 1 (LMP1) is involved in PDL1 upregulation. These results were also supported by Chen et al. (45), who demonstrated how EBV(+) lymphomas, including PTLD, express detectable PDL1. In lymphomas, genomic amplification or EBV infection causes the PD-1/PDL signaling pathway activation with the immune surveillance escape (Figure 1).

The distinctive copy number alteration in EBV(+) PTLD was identified as a gain of 9p21 with respect to EBV(–) PTLD. Gain of 9p21 caused different CDKN2A expression. CDKN2A codes for cyclin-dependent kinase inhibitor 2A (p16INKA), an important regulator of the cell cycle; in particular, it decelerated cell progression through the G1 phase (48). In EBV(+) PTLD, immunohistochemistry (IHC) demonstrated that a gain of 9p21 was associated with exclusively cytoplasmic expression of the p16INKA protein. The p16INKA seems to be implicated in alternative oncogenic pathways and not as a tumor suppressor in EBV(+) PTLD (48, 49).

A gain of chromosome 3/3q was found in EBV(–) PTLD, and it was absent in EBV(+) PTLD. This alteration caused increased expression of FOXP1 in EBV(–) PTLD; these data were confirmed by QRT-PCR and IHC. FOXP1 encodes a transcriptional regulator implicated in different biological processes and in B-cell lymphomas pathogenesis; and it seems to play a critical role in the pathogenesis of EBV(–) PTLD. However, the connection between EBV infection and FOXP1 is uncertain because EBV downregulates FOXP1 in normal B cells (50–54).

IC-DLBCL has many points in common with EBV(–) PTLD. EBV(–) and EBV(+) PTLD demonstrated different genomic and gene expression profiles. In particular, GEP differences in EBV(+) and EBV(–) PTLD involve inflammation and immune response pathways (31), supporting the hypothesis that the EBV infection has a major impact on the gene expression and alterations in EBV(+) PTLD. On the other hand, the EBV(–) PTLD appears to be more similar to *de novo* lymphomas arising in transplanted patients.

Many studies support the role of cytokines in the pathogenesis of EBV(+) PTLD (55). This hypothesis is supported by the detection of IL-10 transcripts in PTLD biopsies. B-cell lymphomas isolated from EBV(+) PTLD produce IL-10 in a constitutive way and use it as an autocrine growth factor (56). For this reason, serum IL-10 has been proposed as an early marker of PTLD (57–60). It is unclear why IL-10 is altered in EBV(+) PTLD.

EBV infection modifies microRNA expression. Gene arrays demonstrate different microRNA profiles in EBV(+) B-cell lymphoma lines from patients with PTLD, as compared with *in vitro* generated EBV(+) lymphoblastoid cell lines or normal B cells. In particular, microRNA-194 (33) was found to be suppressed in EBV(+) PTLD. MicroRNA-194 overexpression increases apoptosis of EBV(+) B-cell lymphoma lines and attenuates IL-10 production. EBV seems to suppress microRNA-194 in order to increase IL-10 expression. Therefore, microRNA-194 may constitute a new approach to inhibiting proliferation of EBV(+) B-cell lymphomas in PTLD.

## Conclusions and Prospective

In recent years, increasing understanding of the biologic and molecular PTLD pathogenesis has resulted in new therapeutic approaches and improved outcomes for these patients. Although the prognosis of EBV(+) in comparison with EBV(–) PTLD is not clear, frontline therapy in EBV(+) and EBV(–) PTLD is currently the same.

In this work, we reported much evidence that EBV(+) and EBV(–) PTLD have distinct genomic and transcriptomic landscape, although at the moment, clinical data do not completely support this hypothesis. EBV(–) PTLD and IC-DLBCL seem to be similar biological entities; for this reason, EBV(–) PTLD might be considered as a type of lymphoma that develops coincidentally in transplant recipients. Moreover, EBV(+) PTLD and EBV(+) DLBCL present many similarities, indicating that EBV both infection and reactivation have important consequence on their pathogenesis (30–32, 38).

PTLD therapy is a combination of reduction of immunosuppressive therapy, immunotherapy, and chemotherapy (23, 25). In this review, we summarize the clinical and biological differences of EBV(+) and EBV(–) PTLD, and we support a new therapeutic approach based on EBV status to improve outcomes of these patients.

The expression of viral antigens makes EBV(+) PTLD an attractive candidate for specific therapy. Unfortunately, latent EBV-infected B cells do not express EBV-thymidine kinase transcript/protein; and for this reason, they are unaffected by antiviral agents as purine nucleoside analog. Similarly, EBV-related lymphoproliferative disorders do not express viral protein kinase, and so monotherapy with nucleoside analogs failed to induce responses in EBV(+) PTLD. However, pharmacological induction of viral thymidine kinase by the administration of the histone deacetylase inhibitor arginine butyrate, followed by antiviral therapy, has shown promising results with an acceptable toxicity profile (61).

More recently, several studies demonstrated how immunomodulatory drugs such as lenalidomide or proteasome inhibitors, in particular bortezomib, can induce EBV lytic activation (62, 63).

The search for new antivirals is ongoing; in particular, a new antiviral agent hexadecyloxypropyl-cidofovir (HDP-CDV) exhibits a remarkable increase in antiviral activity *in vitro* against different double-stranded DNA viruses including EBV (64).

Constitutive activation of the PI3K/Akt/mTOR pathway was shown in *in vitro* EBV(+) PTLD cell lines. Inhibition of either Akt or PI3K, with specific inhibitors CAL-101 or MK-2206, respectively, suppresses EBV(+) PTLD cell growth; and the combination of rapamycin had a synergistic effect. The combination therapy with an Akt inhibitor, or a PI3K inhibitor, and rapamycin can be an efficacious treatment for EBV(+) PTLD (37).

Most results presented are based on *in vitro* data; further evaluation and prospective clinical trials are necessary before such agents can be used as a treatment for PTLD patients.

The upfront treatment of EBV(+) and EBV(–) PTLD is the same, except for the use of EBV-specific adoptive immunotherapy. Immune-based therapies are an effective approach because of EBV antigen expression. In particular, adoptive therapy is based on the high efficacy of unselected donor lymphocyte infusions in HSC transplantation PTLD (65). Attempts were made to isolate EBV-specific cytotoxic lymphocytes (CTLs) aiming to induce a strong EBV-specific cellular immune response without the risk of graft-vs.-host disease (GVHD). Both autologous and allogeneic [isolated from the donor itself or a partial human leukocyte antigen (HLA)-matched donor] CTLs, targeting specific immunogenic EBV antigens, can be used (66). In a large multicentric study, HSCT patients were treated with EBV-CTLs, either prophylactically or therapeutically (67). A Chinese prospective study in HSCT recipients demonstrated an increase in complete remission rates in patients treated with sequential administration of rituximab and EBV-CTLs (68).

Moreover, checkpoint inhibition seems to be a potential treatment option in EBV(+) PTLD. EBV infection/reactivation causes a cytotoxic T-cell dysfunction in lymphomas as PTLD and classical HL. EBV causes an upregulation of immune checkpoint markers. In classical HL, immune checkpoint inhibitors have demonstrated efficacy; and therefore, there has been an increasing interest in PTLD (69). Antigen-presenting cells express PD-L1 that bind the PD-1 receptor on T cells, thus inhibiting T-cell receptor functions. EBV plays a role in increasing PD-L1; these data support the role of checkpoint inhibition in PTLD (44). Kinch et al. demonstrated than PDL-1, PDL-2, and PD-1 were positive in more than half of PTLD cases following SOT (70). More clinical data are necessary to determine the safety, efficacy, and graft rejection risk or GVHD of immune checkpoint inhibitors in PTLD. Currently, a phase II trial (NCT03258567) of nivolumab in a cohort of patients—EBV(+) non-HLs including EBV(+) PTLD—is ongoing.

This review summarizes many steps that have been made in understanding the EBV(+)/(–) PTLD biology. The biological differences connected with the EBV status support the development of preventive/preventive strategies against EBV disease and implementation of existing therapies both in the frontline and in the setting of relapsed/refractory patients. Several molecular targeting agents including immunomodulatory agents, proteasome inhibitors, PI3K and Akt inhibitors, novel anti-CD20 monoclonal antibodies, and immune checkpoint inhibitors seem to have a therapeutic potential, providing a strong rationale for new clinical trials to improve the outcome of EBV post-transplant lymphoproliferative disorder.

## Author Contributions

All authors listed have made a substantial, direct and intellectual contribution to the work, and approved it for publication.

## Conflict of Interest

The authors declare that the research was conducted in the absence of any commercial or financial relationships that could be construed as a potential conflict of interest. The reviewer LF declared a past co-authorship with author LB to the handling Editor.
